# Breaking dependency: The Cinderella complex and barriers to self-employment among rural women in Iran

**DOI:** 10.1371/journal.pone.0337652

**Published:** 2026-02-25

**Authors:** Zahra Seyedtabatabaei, Mahsa Fatemi

**Affiliations:** Department of Agricultural Extension and Education, School of Agriculture, Shiraz University, Shiraz, Iran; Pingdingshan University, CHINA

## Abstract

Despite increasing global emphasis on gender equality, rural women in patriarchal societies continue to face complex and multifaceted barriers to economic participation, particularly in self-employment. This study investigates the psychological and socio-structural dynamics influencing self-employment intentions among rural women in Fars Province, Iran, with a particular focus on the Cinderella complex—a psychological pattern characterized by dependency and fear of independence. Drawing on data from a survey of 365 rural women, collected via a structured questionnaire and analyzed using confirmatory factor analysis and path analysis, the research examines how the Cinderella complex mediates the relationship between structural, cultural, and environmental factors and women’s entrepreneurial behavior. Key psychological constructs—self-efficacy, empowerment, and entrepreneurial enthusiasm—were explored as potential enablers of self-employment. Findings reveal that the Cinderella complex exerts a significant negative effect on self-employment by undermining self-efficacy and reducing entrepreneurial enthusiasm. In contrast, higher levels of self-efficacy and enthusiasm positively influence women’s willingness to engage in entrepreneurial activities. Empowerment also emerged as a critical factor, directly enhancing women’s entrepreneurial engagement. Group comparisons revealed that older women experienced higher independence anxiety, university-educated women reported lower Cinderella complex scores and higher self-efficacy, and employed women demonstrated greater empowerment and self-employment engagement than homemakers. The study underscores the importance of addressing both internal psychological barriers and external structural inequalities to foster self-employment among rural women. It advocates for gender-sensitive policies, empowerment programs, educational initiatives, and community-based support systems to transform traditional gender norms and unlock the entrepreneurial potential of rural women in Iran and similar contexts.

## 1. Introduction

Despite comprising nearly half of the global population, women continue to face significant barriers to economic participation—particularly in self-employment [1,2]. This underrepresentation is not solely due to structural inequality but also deeply rooted in psychological and socio-cultural dynamics [3]. In patriarchal societies, traditional norms frequently constrain women’s autonomy, conditioning them from early childhood into roles characterized by dependency, passivity, and limited agency [4,5]. The present study explores how structural, cultural, and environmental factors interact with internalized psychological patterns—specifically, the Cinderella complex—to influence women’s entrepreneurial behavior in rural Iran.

In the context of Iran, rural women face compounded barriers to economic participation due to a combination of patriarchal cultural norms, limited access to formal employment, and regional disparities in education and infrastructure [6–9]. While women constitute a significant share of the agricultural and informal sectors, their contributions often remain undervalued and unsupported by formal institutional frameworks [10]. Gender-based restrictions on mobility, property ownership, and credit access further constrain their economic autonomy, particularly in traditional rural communities [11,12]. As a result, rural women in provinces such as Fars are often forced to rely on familial support or informal labor, reinforcing cycles of dependency and vulnerability [13,14]. These systemic constraints underscore the urgent need for empowering pathways—such as self-employment—that can enhance rural women’s agency, income security, and social status [15–18].

The Cinderella complex, a term coined by [19], describes a subconscious desire for protection and a fear of independence, particularly among women socialized in patriarchal contexts. These women are often portrayed as graceful, polite, and beautiful—yet fundamentally incapable of self-directed action [20]. Such narratives reinforce dependency by glorifying external rescue, often embodied by male figures. This dynamic is amplified through overprotective parenting, which limits autonomy and fosters reliance on others for major life decisions [21]. Women exhibiting high levels of this complex may experience diminished self-confidence, emotional dependence, and reluctance to pursue independent economic activity [22].

Recent empirical and theoretical studies suggest that the Cinderella complex is not merely a personality trait but is shaped and reinforced by cultural and socialization processes [23]. In rural and patriarchal contexts, gendered expectations, social norms, and collective family practices can amplify dependency tendencies, constraining self-efficacy and the motivation to pursue independent economic roles [24]. For instance, studies in developing country contexts demonstrate that women exposed to restrictive social norms show higher internalization of dependency beliefs, reduced entrepreneurial intentions, and lower agency in decision-making [25–27]. Integrating these findings situates the Cinderella effect within a broader, context-dependent framework, providing a culturally sensitive lens to understand its impact on women’s entrepreneurial behavior.

In the context of labor markets, especially in developing countries, this dependency translates into underemployment and economic vulnerability [28]. Women are disproportionately represented in informal, low-wage, or part-time roles and are more likely to depend on family support or government aid [29,30]. Although self-employment offers a pathway to economic independence, flexibility, and innovation [31], psychological barriers—like the Cinderella complex—can prevent women from seizing these opportunities [32,33].

One psychological construct that mediates this dynamic is self-efficacy—defined by Bandura [34,35], as one’s belief in their ability to execute specific tasks to achieve desired outcomes. Self-efficacy refers to task-specific competence, closely tied to persistence, resilience, and the ability to navigate uncertainty [36–38]. Women with high self-efficacy are more likely to pursue self-employment, innovate, and persevere despite setbacks [39]. Yet, in environments where women lack positive role models and receive negative reinforcement, their self-efficacy often remains underdeveloped [40,41].

Closely related is the construct of women’s empowerment, which refers to the expansion of women’s ability to make strategic life choices in a context where this ability was previously denied [42]. Empowerment is multidimensional—encompassing psychological, economic, social, and political domains—and serves as both a process and an outcome [43,44]. Women’s empowerment is central to sustainable development and has been shown to reduce poverty, improve health outcomes, and strengthen community resilience [45,46]. However, gender-based discrimination continues to restrict women’s access to resources such as education, credit, and leadership roles—obstructing both individual development and broader societal progress [47].

Another pivotal psychological construct examined in this study is entrepreneurial enthusiasm—an affect-laden motivational state that energizes and sustains the pursuit of entrepreneurial goals. Enthusiasm is more than emotional positivity; it encompasses cognitive evaluations of opportunity, emotional arousal, behavioral activation, and expressive cues that signal commitment and resilience [48,49]. It functions both as a personal resource and a social signal—reinforcing confidence internally and projecting credibility externally [50,51]. Importantly, entrepreneurial enthusiasm interacts synergistically with self-efficacy, amplifying motivation and facilitating the reframing of obstacles as growth opportunities [52,53].

**Fig 1:**
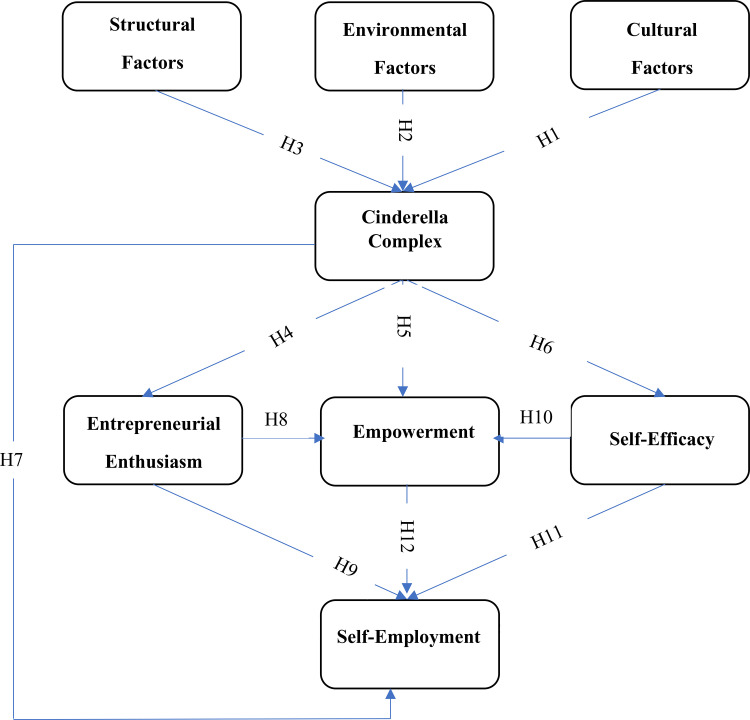
Conceptual framework of the constructs influencing self-employment among rural women.

This study investigates how the Cinderella complex, self-efficacy, empowerment, and entrepreneurial enthusiasm interact to influence women’s decisions regarding self-employment. The research is situated in rural areas of Fars Province, Iran, where gender norms and limited institutional support often constrain women’s economic agency. The conceptual model (Fig 1) positions structural, environmental, and cultural factors as independent variables that shape dependency traits; the Cinderella complex as a primary mediator; and self-efficacy, empowerment, and entrepreneurial enthusiasm as second-level mediators that directly influence women’s willingness to pursue self-employment. To guide this investigation, the following research questions are posed:

***RQ1:*** Do cultural, environmental, and structural factors influence the formation of the psychological index of the Cinderella complex?

***RQ2:*** What are the effects of the Cinderella complex on psychological characteristics of women, such as levels of self-efficacy, empowerment, and entrepreneurial enthusiasm, in the context of self-employment?

***RQ3:*** What are the key factors influencing women’s willingness to engage in self-employment and start their own private business?

## 2. Hypotheses development

The conceptual model of this study draws upon Social Cognitive Theory (SCT) [34,35] and the Theory of Planned Behavior (TPB) [54] to explain the pathways through which contextual and psychological factors influence women’s pursuit of self-employment. Both frameworks highlight the interplay between external conditions and internal cognitions in shaping behavior, which provides a strong theoretical basis for positioning the Cinderella complex, self-efficacy, empowerment, and entrepreneurial enthusiasm as key mediating constructs.

### 2.1. Contextual antecedents and the Cinderella complex

According to SCT, environmental influences play a critical role in shaping cognitive schemas and behavioral tendencies. Cultural values, structural arrangements, and immediate environmental conditions can reinforce or diminish dependency traits in women [35]. Similarly, TPB suggests that normative pressures and perceived control influence women’s attitudes toward autonomy and self-reliance [54]. In patriarchal contexts, cultural and structural systems may either perpetuate reliance on external support or encourage independence, thereby affecting the intensity of the Cinderella complex.

Although Bandura’s work provides a foundational understanding of self-efficacy, subsequent empirical studies highlight that efficacy beliefs are deeply embedded in cultural and socialization processes [55,56]. Research shows that gender norms, power hierarchies, and collective expectations significantly shape how women evaluate their capabilities, particularly in conservative or rural environments [57]. Women exposed to restrictive social norms often report lower efficacy appraisals, limited perceived agency, and a stronger reliance on external authority figures—patterns that closely mirror dependency-oriented dispositions [58]. Integrating these findings strengthens the argument that contextual factors play a decisive role in shaping the cognitive foundations that underlie the Cinderella complex.

***H1:*** *Cultural factors negatively influence the formation of the Cinderella complex, such that societies with progressive norms reduce women’s dependency tendencies.*

***H2:*** *Favorable environmental conditions, including supportive family and educational settings, are associated with a lower intensity of the Cinderella complex among women.*

***H3:*** *Structural factors, such as institutional arrangements and access to resources, significantly shape the extent to which women internalize dependency patterns characteristic of the Cinderella complex.*

### 2.2. Cinderella complex and psychological mediators

The Cinderella complex reflects internalized dependency and fear of independence [19]. From an SCT perspective, such beliefs constrain women’s self-efficacy by limiting their confidence in their ability to pursue entrepreneurial activities. Likewise, TPB emphasizes that negative attitudes and perceived lack of control undermine motivation toward goal-directed behavior [59,60]. Thus, the Cinderella complex is expected to suppress entrepreneurial enthusiasm, self-efficacy, and empowerment, which serve as critical psychological precursors to self-employment. Moreover, the Cinderella complex can be conceptualized as a significant psychological barrier within entrepreneurial contexts, as it aligns with broader theories of gendered socialization that emphasize dependency and conformity over autonomy and initiative [22]. By shaping women’s beliefs about their own capacities and roles, it indirectly weakens the motivational and cognitive foundations necessary for entrepreneurial action [61]. These dynamics highlight the importance of examining the Cinderella complex not only as an intrapersonal constraint but also as a structural impediment that interacts with cultural expectations to shape women’s economic behavior.

While SCT provides the overarching theoretical framework, integrating the Cinderella effect requires attention to cultural and socialization mechanisms. Empirical research indicates that the effect predicts patterns of dependency, risk aversion, and delayed agency that are conceptually aligned with our study’s hypotheses [62]. For example, prior work has shown that women socialized in protective and patriarchal environments exhibit lower self-efficacy, diminished empowerment, and reduced entrepreneurial enthusiasm, paralleling the pathways outlined in H4–H7. By explicitly linking these culturally embedded mechanisms to self-efficacy and entrepreneurial outcomes, the study captures the context-dependent nature of the Cinderella effect, particularly in rural Iranian settings [63]. This theoretical contextualization strengthens the rationale for examining the Cinderella complex as both a psychological and socio-cultural construct influencing women’s entrepreneurial behavior.

***H4:*** *A stronger Cinderella complex diminishes women’s entrepreneurial enthusiasm, as internalized dependency undermines motivation to pursue independent economic activity.*

***H5:*** *Women exhibiting higher levels of the Cinderella complex experience lower empowerment, reflecting reduced agency and control over personal and economic decisions.*

***H6:*** *The Cinderella complex negatively impacts self-efficacy, limiting women’s confidence in their ability to achieve entrepreneurial and self-directed goals.*

***H7:*** *Higher levels of the Cinderella complex reduce the likelihood of women engaging in self-employment, reflecting the behavioral consequences of dependency tendencies.*

### 2.3. Psychological resources and self-employment

SCT underscores the importance of self-efficacy in driving persistence and resilience in the face of challenges [34]. Women with higher self-efficacy are more likely to engage in proactive economic behavior and resist dependency. Entrepreneurial enthusiasm, as an affective-cognitive state, further strengthens this pathway by energizing individuals toward goal pursuit [64]. Empowerment, reflecting women’s agency and control over resources, emerges as both a direct outcome of these psychological resources and a critical mediator that channels them into entrepreneurial behavior.

***H8:*** *Entrepreneurial enthusiasm positively influences women’s empowerment, as motivated engagement in entrepreneurial activities strengthens agency and decision-making capacity.*

***H9:*** *Women with greater entrepreneurial enthusiasm are more likely to pursue self-employment, driven by increased motivation and goal-directed behavior.*

***H10:*** *Higher self-efficacy enhances empowerment by enabling women to exert greater control over resources and assert influence in personal and professional domains.*

***H11:*** *Women with stronger self-efficacy are more likely to engage in self-employment, as confidence in one’s capabilities translates into entrepreneurial action.*

***H12:*** *Empowerment positively affects self-employment, with women possessing greater agency and social control demonstrating higher propensity to establish independent ventures.*

The SCT and TPB provide a coherent framework for linking contextual antecedents, dependency-related cognitions, and psychological mediators to women’s entrepreneurial behavior. SCT explains how environmental and cultural factors shape internal cognitions such as the Cinderella complex and self-efficacy, while TPB emphasizes how attitudes, subjective norms, and perceived behavioral control influence enthusiasm, empowerment, and ultimately self-employment (Fig 1).

## 3. Research method

This survey study targeted all rural women residing in Fars Province, Iran, as the statistical population. Based on Krejcie and Morgan [65], a total of 384 individuals were randomly selected as the sample. Considering a 95% response rate, the final sample consisted of 365 rural women from six counties and 52 villages across Fars Province. Data were collected through a structured questionnaire, developed based on previous studies. The face validity of the instrument was confirmed by faculty members at Shiraz University. The data collection was conducted in person over a five-month period, From 27 November 2024 to 22 April 2025, with detailed explanations provided to all participants. This study was reviewed and approved by the Higher Education Committees at both the School of Agriculture and Shiraz University, ensuring adherence to ethical standards. The approval was issued under reference number 3GCB2M224945, dated 2024-11-23. All participants provided written informed consent before taking part in the study. We confirm that all research procedures were conducted in accordance with the relevant ethical guidelines and regulations for research involving human participants, as outlined by the Higher Education Committees at Shiraz University and in compliance with internationally recognized ethical principles. The authors did not access any information that could identify individuals during or after data collection.

The questionnaire was designed to comprehensively cover all dimensions of the study and to measure the relevant research variables. Items were structured using 5 item Likert-type scales depending on the variable. In the initial phase, descriptive statistics of the respondents and mean comparison tests (ANOVA) were conducted and summarized using SPSS26. Subsequently, confirmatory factor analysis (CFA) was employed using SmartPLS3 software to assess the reliability and validity of the research constructs, based on Cronbach’s alpha, composite reliability (CR), convergent validity (average variance extracted – AVE), and discriminant validity. Finally, in the last stage, the data were analyzed using path analysis through AMOS_24_.

### 3.1. Research variables

Environmental factors in this study pertain to two key dimensions: parenting styles within the family context and instructional styles experienced in educational institutions such as schools and universities. Parenting style refers to the overall emotional climate of the parent-child relationship, encompassing specific patterns of interaction between parents and their children. In terms of instructional style, emphasis is placed on pedagogical methods, learning strategies, assessment techniques, and the management of educational processes—factors that collectively influence academic readiness and student development [66,67]. This variable was measured using 14 items on a five-point Likert scale ranging from “Strongly Disagree” to “Strongly Agree.” These items capture women respondents’ perceptions of broader gendered practices and social norms in familial and educational environments.

Structural factors refer to broader socio-institutional elements, including social differentiation, patterns of production and labor, systems of communication, levels of functional autonomy, institutional roles, and the distribution of social positions [68]. These factors represent foundational components that shape individual and group behavior within society. In the present study, structural factors were assessed using 8 items rated on a five-point Likert scale, from “Strongly Disagree” to “Strongly Agree.” The items reflect women respondents’ perceptions of institutional and structural constraints affecting gender equality and opportunities within their social context.

Cultural factors encompass values, beliefs, norms, customs, legal traditions, and symbolic elements that characterize a society’s collective identity. Culture is constituted by the shared attitudes, ideologies, and behavioral expectations of social groups, while also allowing for individual variation in interpretation and expression [69]. This variable was measured using 10 items on a Likert scale ranging from “Not at All” to “Very Much.” These items are designed to capture women respondents’ perspectives on societal gender norms, expectations, and cultural practices that shape their experiences and opportunities.

The Cinderella complex—defined as a hidden fear of independence among women—is a psychological condition marked by a subconscious desire to be cared for and rescued, often symbolized by the archetype of a “prince on a white horse” [19]. In this study, the Cinderella complex was operationalized through five distinct dimensions: Independence Anxiety (IA), Avoidance of Responsibility (AR), Unrealistic Expectations (UE), Desire for Uniqueness (DU), and Validation Seeking (VS). These were measured using 44 items on a five-point Likert scale ranging from “Strongly Disagree” to “Strongly Agree.” These items are based on women respondents’ self-assessments of their personal psychological tendencies and beliefs, reflecting individual personality traits related to dependency and autonomy.

Drawing from the literature in organizational psychology, Entrepreneurial enthusiasm is conceptualized as a specific form of positive emotional energy oriented toward future engagement in goal-driven activities, including business creation and entrepreneurial pursuits [70]. It represents both an emotional and motivational construct that supports initiative and persistence. In this study, entrepreneurial enthusiasm was evaluated using 6 items, each rated on a five-point Likert scale from “Strongly Disagree” to “Strongly Agree.” The items reflect women respondents’ personal self-assessments of motivation, interest, and enthusiasm toward entrepreneurial activities.

Empowerment is understood as a multidimensional social process through which individuals gain control over their lives, enabling them to take action on matters they deem important. Empowerment facilitates individual and collective capacity building at various levels: personal, community, and societal [71,72]. In this study, empowerment was examined across four interrelated dimensions. The economic dimension involves providing individuals with the tools and resources necessary for participation in economic life, access to financial opportunities, and achievement of financial independence [73]. The social dimension encompasses the social structures, relationships, and processes that enable individuals to engage meaningfully in their environments and enhance their capacity to act [74]. The psychological dimension refers to internal cognitive and emotional processes that cultivate self-confidence, perceived competence, and the belief in one’s ability to influence personal and professional outcomes [75]. Finally, the political dimension addresses individuals’ engagement with governance structures, including access to political information, participation in voting, advocacy, civic activities, and representation at various levels of decision-making [76]. Empowerment was measured using 21 items across these dimensions, with responses recorded on a five-point Likert scale ranging from “Never” to “Always.” The items capture women respondents’ self-perceptions of their personal empowerment and agency across economic, social, psychological, and political domains.

Self-efficacy is defined as an individual’s belief in their capacity to successfully perform specific tasks and attain desired outcomes. Whether in academic, personal, or professional contexts, self-efficacy plays a crucial role in enabling individuals to persist in the face of challenges, overcome adversity, and strive toward excellence [77]. In the current study, self-efficacy was assessed using 9 items measured on a five-point Likert scale ranging from “Strongly Disagree” to “Strongly Agree.” These items reflect women respondents’ self-assessments of their personal confidence and perceived ability to achieve desired outcomes.

Self-employment refers to a form of economic activity in which individuals derive part or all of their income through business ventures they own and operate independently, rather than through salaried employment under an external employer [78]. In this study, self-employment was measured using two sets of items: four questions using a five-point Likert scale ranging from “Very Low” to “Very High,” and seven questions rated from “Strongly Disagree” to “Strongly Agree.” It should be noted that in this study, self-employment captures respondents’ attitudes, perceptions, and intentions toward engaging in entrepreneurial activity, rather than their actual current employment status. The items capture women respondents’ self-reported entrepreneurial activity, personal engagement in business, and independent income-generating behaviors.

## 4. Results

### 4.1. Demographic overview of the sample

The study sample consisted of 365 rural women from six counties in Fars Province, Iran. To provide a qualitative profile of rural women and categorize respondents based on age and educational attainment, the Interval Standard Deviation from the Mean (ISDM) technique was employed. Recognized as a widely accepted method for qualitative categorization of research variables [79], ISDM segments the obtained scores into three distinct levels, as outlined in Table 1. Accordingly, respondents were classified into three age groups: young, middle-aged, and older adults.

**Table 1:** Frequency distribution of respondents in terms of demographic features.

Variables	Groups	Frequency	Percent	Cumulative Percent
Age	Young	51	14	14
	Middle-aged	173	47.4	61.4
	Elderly	137	37.5	98.9
	No answer	4	1.1	100
	Total	365	100	
	*Max: 71*	*Min: 15*	*SD: 9.983*	*Mean: 37.62*
Education	Primary education	12	3.3	3.3
	Secondary education	133	36.4	39.7
	Higher education	218	59.7	99.5
	No answer	2	0.5	100
	Total	365	100	
	*Max:0*	*Min: 25*	*SD: 3.560*	*Mean: 14.23*
Marital status	Single	91	24.9	24.9
	Married	255	69.9	94.8
	Self-Supported	18	4.9	99.7
	No answer	1	0.3	100
	Total	365	100	
Employment	Public employee	81	22.2	22.2
	Self-employed	113	31	53.2
	Unemployed	169	46.3	99.5
	No answer	2	0.5	100
	Total	365	100	
county of residence	Zarghan	63	17.3	17.3
	Marvdasht	61	16.7	34
	Kavar	60	16.4	50.4
	Kazeroun	61	16.7	67.1
	Sarvestan	60	16.4	83.5
	Jahrom	60	16.4	100
	Total	365	100	

As shown in Table 1, the youngest respondent was 15 years old and the oldest was 71, with an average age of 37.62 years. The highest frequency belonged to the middle-aged group (ages 26–40), accounting for 47.4% of the sample. The older adult category (ages 41–71) comprised 37.5%, while the youngest group (ages 15–25) represented 14% of the sample. In terms of education level, Table 1 indicates that the majority of respondents held university degrees, with 218 individuals (59.7%), followed by 133 respondents (36.4%) with secondary education, and 12 participants (3.3%) who were illiterate or had only completed primary education. Although the proportion of university-educated women may appear high for a rural context, national statistics indicate substantial progress in female education in rural Iran over the past decades. Rural literacy rates increased from 30.5% in 1976 to 78.5% in 2016 [80], and women have consistently comprised the majority of university entrants in recent years [81], accounting for nearly 48% of the student population in 2018–2019 [82]. These trends explain the high representation of university-educated women in the current sample.

The respondents, based on marital status, were categorized into single, married, and female heads of household (widowed or divorced). The majority were married (69.9%), followed by single (24.9%) and female heads of household (4.9%). Regarding employment status, women were divided into three groups: employed in the public sector (22.2%), engaged in private businesses (31%), and unemployed or homemakers (46.3%). Notably, despite the high proportion of university-educated women, a substantial share remained unemployed or homemakers, reflecting the persistently low female labor force participation and structural barriers to employment in rural areas. Respondents were drawn from six counties in Fars Province—Zarqan, Marvdasht, Kavar, Kazeroon, Sarvestan, and Jahrom—each contributing approximately 60 participants.

### 4.2. Comparison of rural women by age group

First, the respondents were compared based on age groups with respect to the study variables. Participants were categorized into three age groups: young, middle-aged, and older women. According to the results, no significant differences were observed between age groups in terms of the overall Cinderella complex.

However, the analysis of variance (ANOVA) results, presented in Table 2, indicated a significant difference among the three age groups for the independence anxiety component (P = 0.023). According to the LSD post-hoc test, this difference was observed between the young and middle-aged groups compared with the older group. Specifically, the mean score for independence anxiety was higher in older women (42.36) compared to middle-aged (38.58) and young women (37.57), suggesting that concerns about independence, as a component of the Cinderella complex, increases with age. Comparisons of the other Cinderella complex components—avoidance of responsibility**,** unrealistic expectations**,** desire for uniqueness**,** and validation seeking—did not reveal significant differences across the three age groups (Table 2).

**Table 2:** Comparison of rural women across age groups on study variables.

Variables	Elderly		Middle-aged		Young		F	Sig.
	Mean	SD	Mean	SD	Mean	SD		
Cinderella Complex	101.67	21.671	101.51	24.822	106.55	27.207	1.661	0.191
Independence Anxiety	37.57a	12.166	38.58a	12.652	42.36b	15.061	3.828	0.023
Avoidance of Responsibility	25.43	6.175	45.54	7.446	26.12	7.924	0.284	0.753
Unrealistic Expectation	8.47	2.444	8.43	2.604	8.8	2.712	0.797	0.452
Desire for Uniqueness	12.14	3.682	11.75	3.77	11.35	3.187	1.041	0.354
Validation Seeking	18.06	4.768	17.21	5.351	17.93	5.594	0.901	0.407
Self-Efficacy	32.08	5.298	33.75	5.606	33.97	5.676	2.249	0.107
Empowerment	36.45	8.484	37	9.995	38.69	11.357	1.368	0.256
Economic Empowerment	8.53	3.722	9.02	3.918	9.32	4.602	0.689	0.503
Social Empowerment	8.04	2.049	8.22	2.369	8.5	2.503	0.871	0.419
Psychological Empowerment	15.98	3.569	16.07	4.612	16.26	4.595	0.098	0.907
Political Empowerment	3.90a	2.202	3.69a	2.201	4.62b	2.259	6.898	0.001
Entrepreneurial Enthusiasm	21.71	3.823	21.78	4.859	21.71	4.408	0.012	0.989
Self-Employment	36.53	4.851	36.86	6.423	35.31	6.507	2.428	0.09
Cultural Factors	19.86	5.903	19.28	6.508	18.82	5.93	0.565	0.569
Environmental Factors	36.71	6.008	35.87	7.683	35.68	7.966	0.347	0.707
Parenting Style	17.33	3.824	17.27	4.119	17.28	4.521	0.004	0.996
Instructional Style	19.37	3.555	18.6	4.53	18.4	4.4	0.935	0.394
Structural Factors	26.43	4.579	27.35	5.854	27.23	5.35	0.558	0.573

*Means sharing the same letter within a row are not significantly different at the 0.05 level.*

**Variables` range**: Cinderella Complex (42–210), Independence Anxiety (17–85), Avoidance of Responsibility (10–50), Unrealistic Expectations (3–15), Desire for Uniqueness (5–25), Validation Seeking (7–35), Self-Efficacy (9–45), Empowerment (0–64), Economic Empowerment (0–20), Social Empowerment (0–12), Psychological Empowerment (0–24), Political Empowerment (0–8), Entrepreneurial Enthusiasm (6–30), Self-Employment (9–45), Cultural Factors (8–40), Environmental Factors (12–60), Parenting Style (6–30), Instructional Style in School and University (6–30), Structural Factors (7–35).

In addition, the variable empowerment and its components were examined across the three age groups. ANOVA results (Table 2) showed a significant difference for the political empowerment component (P = 0.001). Post-hoc comparisons indicated that older women had a significantly higher mean score for political empowerment (4.62) compared to middle-aged (3.69) and young women (3.90). This suggests that older women are politically more empowered than their younger counterparts.

### 4.3. Comparison of rural women by educational level

The means of the study variables were compared across three educational groups: primary education, secondary education, and higher education (Table 3). ANOVA results revealed a significant difference among educational groups for the Cinderella complex (P = 0.0001). Post-hoc LSD tests indicated that this difference was present across all three groups, with university-educated women reporting the lowest mean score (97.99) and women with primary education the highest (127.17). These results suggest that higher education, particularly at the university level, is associated with a reduction in the Cinderella complex.

**Table 3:** Comparison of study variables among rural women across educational levels.

Variables	Higher Education		Secondary Education		Primary Education		F	Sig.
	Mean	SD	Mean	SD	Mean	SD		
Cinderella Complex	127.17a	28.651	110.90b	27.371	97.99c	21.689	17.678	0.0001
Independence Anxiety	52.67a	17.259	43.54b	15.281	37.11c	11.265	15.809	0.0001
Avoidance of Responsibility	31.17a	9.154	27.32a	7.443	24.63b	7.02	9.013	0.0001
Unrealistic Expectation	11.00a	2.045	9.28b	2.603	8.04c	2.5	15.539	0.0001
Desire for Uniqueness	11.25	2.768	12.08	3.44	11.38	3.619	1.706	0.183
Validation Seeking	21.08a	4.441	18.68a	5.675	16.82b	5.004	7.88	0.0001
Self-Efficacy	30.33b	6.02	33.03ab	5.827	34.17a	5.35	3.941	0.02
Empowerment	29.00a	9.371	35.32b	10.925	39.45c	9.502	11.554	0.0001
Economic Empowerment	5.58a	3.605	7.95a	3.764	9.92b	4.167	14.563	0.0001
Social Empowerment	7.25a	2.417	7.95a	2.612	8.57b	2.176	4.112	0.01
Psychological Empowerment	12.33a	4.292	15.33b	4.92	16.82c	3.99	9.51	0.0001
Political Empowerment	3.83	2.725	4.08	2.286	4.15	2.214	0.128	0.879
Entrepreneurial Enthusiasm	18.67a	4.539	22.38b	4.471	21.57b	4.497	4.299	0.01
Self-Employment	31.58a	5.485	36.10b	6.39	36.60b	6.138	3.764	0.024
Cultural Factors	15.17a	4.783	17.89a	6.062	20.21b	6.13	8.761	0.0001
Environmental Factors	39.83	12.134	36.61	7.982	35.33	6.915	2.855	0.059
Parenting Style	20.25a	6.298	17.74b	4.54	16.88b	3.829	4.823	0.009
Instructional Style	19.58	6.667	18.86	4.334	18.45	4.208	0.669	0.513
Structural Factors	24.75	6.283	27.59	4.999	27.06	5.658	1.632	0.197

*Means sharing the same letter within a row are not significantly different at the 0.05 level.*

**Variables` range**: Cinderella Complex (42–210), Independence Anxiety (17–85), Avoidance of Responsibility (10–50), Unrealistic Expectations (3–15), Desire for Uniqueness (5–25), Validation Seeking (7–35), Self-Efficacy (9–45), Empowerment (0–64), Economic Empowerment (0–20), Social Empowerment (0–12), Psychological Empowerment (0–24), Political Empowerment (0–8), Entrepreneurial Enthusiasm (6–30), Self-Employment (9–45), Cultural Factors (8–40), Environmental Factors (12–60), Parenting Style (6–30), Instructional Style in School and University (6–30), Structural Factors (7–35).

Further analyses of the components of the Cinderella complex revealed significant differences for independence anxiety (P = 0.0001), with women holding primary education scoring highest (52.67), followed by secondary education (43.54), and university-educated women the lowest (37.11). This finding indicates that higher education fosters greater independence among women. Similarly, avoidance of responsibility and unrealistic expectations exhibited significant differences (P = 0.0001), with university-educated women demonstrating lower avoidance of responsibility (24.63) and lower unrealistic expectations (8.04) than the other groups, suggesting that higher education promotes responsibility and realistic goal-setting. No significant differences were observed for desire for uniqueness, whereas validation seeking differed significantly, with university-educated women showing the lowest mean (16.82), indicating less reliance on external validation.

Self-efficacy also varied significantly across educational levels (P = 0.020). Post-hoc comparisons showed that university-educated women reported higher self-efficacy (34.17) than women with primary education (30.33), while no significant differences were observed between secondary education and the other groups. Analysis of empowerment and its components revealed significant differences for overall empowerment (P = 0.0001), with women with primary education reporting the lowest mean (29.0), secondary education (35.32), and university-educated women the highest (39.45). These results indicate that higher education enhances women’s ability to address challenges and exercise control in personal and social contexts. Specifically, economic empowerment was highest among university-educated women (9.92). Significant differences were also observed in Social Empowerment (P = 0.01) and psychological empowerment (P = 0.0001), with university-educated women scoring highest. No significant differences were found for Political Empowerment.

Regarding entrepreneurial enthusiasm, ANOVA indicated significant differences among educational groups (P = 0.01). Women with secondary (22.38) and higher education (21.57) demonstrated significantly higher enthusiasm than women with primary education (18.67). Similarly, self-employment differed significantly (P = 0.024), with university-educated women showing greater intention and engagement in independent business activities, reflecting stronger entrepreneurial attitudes and self-reported business behaviors. For cultural factors, significant differences were observed (P = 0.0001), with university-educated women scoring highest (20.21). In contrast, environmental factors and instructional style in school and university did not differ significantly across groups. Analysis of parenting style revealed significant differences (P = 0.009), with primary-educated women reporting the highest mean (20.25). No significant differences were found for Structural Factors.

These findings align with previous research demonstrating that education is a critical determinant of gender equality and women’s empowerment [61]. Higher education equips women with the knowledge, skills, and opportunities necessary for personal and professional development, while limited education can impose economic and social constraints. Education is therefore essential for fostering economic development and enhancing women’s capacity to participate in and benefit from societal growth [83]. Furthermore, individuals with higher education and greater resources are more likely to engage in self-employment, particularly in agricultural sectors [84].

### 4.4. Comparison of rural women by employment status

ANOVA results (Table 4) indicated significant differences among the three employment groups regarding the Cinderella complex (P = 0.0001). Post-hoc LSD tests revealed that this difference was observed between homemakers and women employed in either the public or private sector, with homemakers exhibiting the highest mean score (111.85) compared to public-sector employees (96.42) and private-sector employees (96.41). These findings suggest that employment, whether in the public or private sector, is associated with a lower level of the Cinderella complex, whereas homemakers display higher levels.

**Table 4:** Comparison of study variables among rural women by employment status.

Variables	Unemployed		Self-employed		Public employee		F	Sig.
	Mean	SD	Mean	SD	Mean	SD		
Cinderella Complex	96.42a	24.306	96.41a	19.381	111.85b	27.077	18.224	0.0001
Independence Anxiety	37.32a	11.475	36.65a	11.348	43.42b	15.189	10.816	0.0001
Avoidance of Responsibility	23.60a	7.628	24.09a	6.377	28.03b	7.484	15.092	0.0001
Unrealistic Expectation	7.06a	2.532	7.96a	2.407	9.27b	2.659	11.242	0.0001
Desire for Uniqueness	10.85a	3.575	11.39ab	3.478	12.15b	3.474	4.166	0.016
Validation Seeking	16.58a	5.201	16.33a	4.388	18.97b	5.739	10.678	0.0001
Self-Efficacy	34.74a	5.125	35.14a	4.998	32.11b	5.796	2.759	0.0001
Empowerment	42.53a	9.517	38.04b	8.988	34.97c	10.658	16.075	0.0001
Economic Empowerment	11.63a	3.929	9.46b	3.732	7.53c	3.873	13.179	0.0001
Social Empowerment	8.83a	2.126	8.64a	1.964	7.84b	2.635	6.524	0.002
Psychological Empowerment	17.49a	3.775	16.19b	4.159	15.48b	4.793	5.773	0.003
Political Empowerment	4.58a	2.15	3.74b	2.039	4.12ab	2.416	3.285	0.039
Entrepreneurial Enthusiasm	21.1	4.491	22.04	4.508	21.95	4.513	0.231	0.293
Self-Employment	34.57a	6.257	38.27b	5.787	35.75a	6.235	9.864	0.0001
Cultural Factors	20.42a	6.551	19.96a	5.641	18.07b	6.228	5.383	0.005
Environmental Factors	35.72	7.415	35.24	6.328	36.57	8.374	1.109	0.331
Parenting Style	17.46	3.908	16.61	3.897	17.73	4.585	2.435	0.089
Instructional Style	18.26	4.398	18.63	3.756	18.84	4.685	0.489	0.613
Structural Factors	26.56	5.566	27.23	5.407	27.58	5.441	0.965	0.382

*Means sharing the same letter within a row are not significantly different at the 0.05 level.*

**Variables` range**: Cinderella Complex (42–210), Independence Anxiety (17–85), Avoidance of Responsibility (10–50), Unrealistic Expectations (3–15), Desire for Uniqueness (5–25), Validation Seeking (7–35), Self-Efficacy (9–45), Empowerment (0–64), Economic Empowerment (0–20), Social Empowerment (0–12), Psychological Empowerment (0–24), Political Empowerment (0–8), Entrepreneurial Enthusiasm (6–30), Self-Employment (9–45), Cultural Factors (8–40), Environmental Factors (12–60), Parenting Style (6–30), Instructional Style in School and University (6–30), Structural Factors (7–35).

Analysis of the independence anxiety component also revealed significant differences (P = 0.0001). LSD tests indicated that homemakers had the highest levels of independence anxiety (43.42) relative to the two employed groups, suggesting that women in the workforce experience greater independence, whereas homemakers are more dependent in managing daily responsibilities. Similarly, the avoidance of responsibility component showed significant differences (P = 0.0001), with homemakers scoring highest (28.03), indicating lower responsibility-taking compared to employed women. For the unrealistic expectations component, ANOVA results demonstrated significant differences among the three employment groups (P = 0.0001). LSD tests showed that homemakers had the highest mean (9.27), suggesting that women who are not employed, tend to have less realistic expectations. A significant difference was also observed for the desire for uniqueness component (P = 0.016), with public-sector employees scoring lower (10.85) and homemakers higher (12.15). The validation seeking component demonstrated significant differences (P = 0.0001), with homemakers reporting the highest mean (18.97), indicating a greater need for external validation.

Regarding empowerment, significant differences were found among employment groups (P = 0.0001), with public-sector employees reporting the highest overall empowerment (42.53), followed by private-sector employees (38.04), and homemakers the lowest (34.97). Economic empowerment showed a similar pattern, with public-sector women scoring highest (11.63), private-sector women intermediate (9.46), and homemakers lowest (7.53). Social empowerment differed significantly among groups (P = 0.002), with employed women more socially empowered (public: 8.83, private: 8.64) than homemakers (7.84). Psychological empowerment also showed significant differences (P = 0.003), with public-sector employees achieving the highest scores (17.49), likely reflecting greater stability and clarity regarding work-related risks. Political empowerment differed significantly between public- and private-sector employees, with public-sector women scoring higher (4.58).

No significant differences were observed among employment groups for entrepreneurial enthusiasm. However, Self-efficacy differed significantly (P = 0.0001), with homemakers reporting the lowest mean (32.11), indicating that employment enhances self-efficacy. Cultural Factors also varied significantly by employment status (P = 0.005), with homemakers scoring lowest (18.07) and public- and private-sector employees higher (20.42 and 19.96, respectively). Regarding self-employment, significant differences were observed (P = 0.0001), with private-sector women exhibiting the highest mean (38.27), suggesting that women employed in the private sector are more inclined toward entrepreneurial engagement. No significant differences were found in Environmental factors or its components (Parenting style in the family; instructional style in school and university) across employment groups. Similarly, structural factors did not vary significantly among the three groups.

### 4.5. Research variables: Validity and reliability

**Fig 2:**
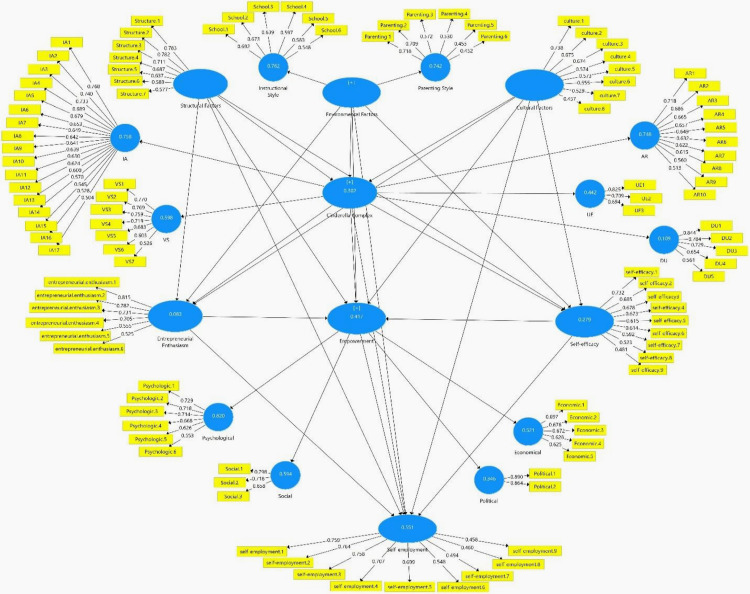
Measurement model of the research variables.

The overall measurement model, aligned with the study’s conceptual framework (Fig 1), was constructed using Smart PLS3 software (Fig 2). The values for factor loadings, composite reliability, and convergent validity of the items related to the key research variables are presented in Table 5. The variable self-efficacy initially consisted of 9 items. All 9 items showed factor loadings above 0.40, confirming their reliability. As reported in Table 5, the Cronbach’s alpha was 0.803, and composite reliability was 0.848—both exceeding the acceptable threshold of 0.70. The AVE value was 0.501, indicating acceptable convergent validity. The variable entrepreneurial enthusiasm consisted of 6 items. As shown in Table 5, all factor loadings exceeded 0.40, confirming acceptable reliability. The Cronbach’s alpha and composite reliability for this construct were 0.779 and 0.842, respectively—both within acceptable limits. The AVE value for this construct was 0.580, demonstrating suitable convergent validity. The variable self-employment initially included 11 items. However, after performing confirmatory factor analysis, 2 items with factor loadings below 0.40 were removed. Ultimately, 9 items remained with acceptable factor loadings, as presented in Table 5. This construct showed strong reliability with a Cronbach’s alpha of 0.813, composite reliability of 0.855, and AVE of 0.507. Each construct demonstrated adequate levels of reliability and convergent validity, supporting the robustness of the measurement model.

**Table 5:** Results of the convergent validity and reliability of self-efficacy, entrepreneurial enthusiasm, self-employment, cultural and structural factors.

Variable	Items	Factor Loading	Cronbach Alpha	CR	AVE
Self-Efficacy	Confidence in achieving plans and goals and putting them into action	0.686	0.803	0.848	0.501
	Giving up and not making an effort when unable to accomplish something for the first time	0.684			
	Ensuring adaptability when encountering unexpected situations such as price fluctuations, natural disasters, the entry of competitors, or similar products, etc.	0.671			
	Ensuring the effective completion of various tasks (such as household chores, work outside the home, caregiving for parents, etc.)	0.663			
	Becoming anxious when facing new and complex phenomena due to doubt about one’s own performance	0.659			
	Focusing on completing a difficult task instead of feeling discouraged	0.642			
	The ability to find more than one solution when facing a problem	0.548			
	The ability to stay calm and rely on one’s abilities when facing problems, even if they are more difficult than expected	0.544			
	Success in choosing any career path	0.457			
Entrepreneurial Enthusiasm	Continuous thinking about entrepreneurship and starting a personal business	0.841	0.779	0.842	0.580
	Great passion for entrepreneurship and personal business	0.777			
	Desire to start a business and daydreaming about it	0.751			
	Enhancement of personal experience through entrepreneurship and starting a business	0.702			
	A sense of identity solely through entrepreneurship and personal business, and the inability to live without it	0.547			
	A willingness to dedicate oneself to entrepreneurial activities	0.465			
Self-Employment	Agreement with registering a business and producing products or offering services based on personal skills, as well as creating employment opportunities for others	0.786	0.813	0.855	0.507
	Achieving all life goals through starting a personal business	0.747			
	Putting in maximum effort to start and manage a personal business	0.741			
	The individual’s willingness to work in an environment where they own the business themselves	0.724			
	Striving to implement ideas through launching a personal business, considering the saturation of public sector jobs	0.699			
	Having sufficient experience to start a personal business	0.557			
	Seeing oneself starting a personal business in the near future	0.478			
	Ability to start a business using personal and family capital	0.452			
	Starting a personal business is preferable due to the waste of valuable talents when working for others	0.426			
Cultural Factors	The acceptance of women’s and girls’ participation in jobs outside the home by society	0.736	0.754	0.817	0.500
	The possibility for girls to travel independently without their family	0.672			
	The possibility of allowing women to pursue education or work after marriage	0.672			
	The possibility of men and women working together in a joint business venture	0.578			
	Equal division of household chores between men and women	0.572			
	The possibility of women being able to stay outside the home and feel safe after dark	0.559			
	The permission for women to seek divorce in case of problems and incompatibility after marriage	0.527			
	The right of women to obtain custody of children after marriage	0.463			
Structural Factors	The greater difficulties and challenges faced by women in accessing loans and financial facilities compared to men	0.784	0.812	0.859	0.570
	The limited access of women to loans, credits, and financial facilities	0.782			
	Greater attention to men’s opinions in work environments	0.713			
	Greater allocation of managerial and executive positions to men	0.687			
	Employers’ preference for hiring male workers	0.639			
	Higher income levels of employed men	0.584			
	Lower job security and stability for women	0.575			

The variable cultural factors were initially measured using 10 items. After conducting the analysis, 2 items with factor loadings below 0.40 were removed, leaving 8 items with acceptable factor loadings (Table 5). The Cronbach’s alpha for this variable was 0.754, confirming its reliability. Additionally, the composite reliability (0.817) and the AVE (0.50) values were also within acceptable thresholds, as shown in Table 5. Regarding structural factors, after confirmatory factor analysis, one item with a factor loading below 0.40 was removed, resulting in a final set of 7 items with acceptable factor loadings. The Cronbach’s alpha for this construct was 0.812, indicating strong internal consistency. The composite reliability was 0.859, and the AVE was 0.570, both demonstrating adequate construct reliability.

One of the criteria for assessing model fit is discriminant validity, which contrasts with convergent validity. In this study, the Fornell and Larcker method [85] was used to evaluate discriminant validity. This type of validity is considered acceptable when the AVE of a latent variable is greater than its correlation with other latent variables. For discriminant validity, a matrix is drawn as shown in Table 6, where the cells contain correlation coefficients between the components, and the diagonal of the matrix contains the square roots of the AVE values for each component. Discriminant validity is considered acceptable when the diagonal values are higher than the values below them. According to Table 6, the latent variables of Cinderella complex with a value of 0.761, cultural factors with a value of 0.707, empowerment with a value of 0.741, entrepreneurial enthusiasm with a value of 0.761, environmental factors with a value of 0.721, self-efficacy with a value of 0.707, self-employment with a value of 0.712, and structural factors with a value of 0.754, all have the highest square root of AVE compared to their corresponding correlations, confirming the discriminant validity of the research model.

**Table 6:** Results of the discriminant validity of research variables.

Variables	Cinderella complex	Cultural factors	Empowerment	Entrepreneurial Enthusiasm	Environmental factors	Self-efficacy	Self-Employment	Structural factors
Cinderella complex	0.761							
Cultural factors	−0.350	0.707						
Empowerment	−0.389	0.412	0.741					
Entrepreneurial Enthusiasm	−0.209	0.186	0.351	0.761				
Environmental factors	0.520	−0.354	−0.173	−0.054	0.721			
Self-Efficacy	−0.508	0.305	0.573	0.368	−0.254	0.707		
Self-Employment	−0.320	0.305	0.379	0.697	−0.133	0.441	0.712	
Structural factors	0.193	−0.212	−0.043	0.086	0.358	−0.118	0.051	0.754

After confirming the validity and reliability of the overall research model, separate confirmatory factor analyses (CFAs) were conducted for three multidimensional constructs—Cinderella Complex, Empowerment, and Environmental Factors—due to their more complex measurement structures. Each construct comprised multiple components, with several items designed for each component. For these constructs, items with low factor loadings were removed, while the retained items demonstrated satisfactory standardized loadings, composite reliability, Cronbach’s alpha values above 0.70, and average variance extracted (AVE) exceeding recommended thresholds, thereby establishing convergent validity. Discriminant validity was also confirmed, indicating that the subcomponents were distinct from one another. Collectively, these results support the robustness of the measurement model for the three constructs. Detailed factor loadings, reliability indices, and discriminant validity matrices are reported in Appendices S1–S3.

### 4.6. Factors influencing the self-employment of rural women: Causal analysis

**Fig 3:**
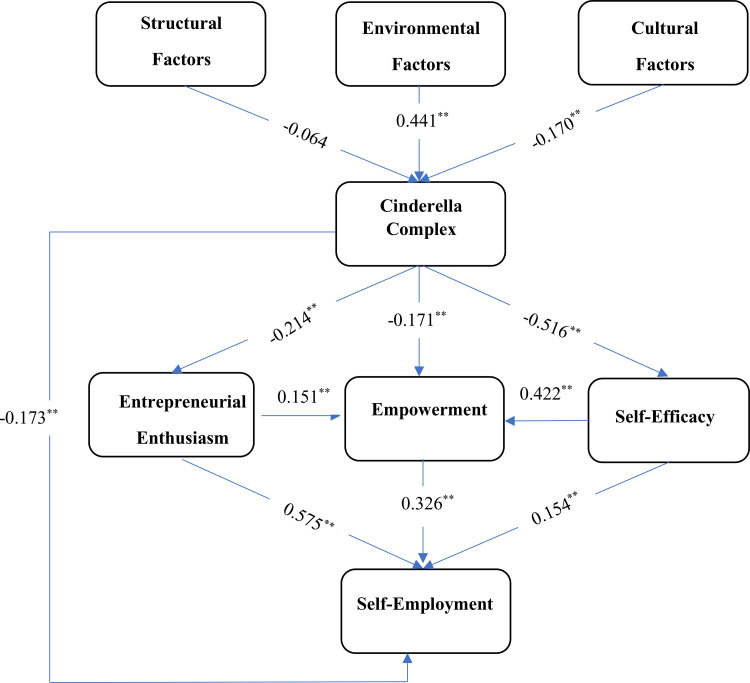
Causal model of factors influencing self-employment among rural women.

The results of the path analysis of the causal model for factors influencing self-employment among rural women are presented in Fig 3. In this analysis, the effects of a set of variables on each other were calculated using AMOS_24_ software, and the standardized path coefficients were obtained. Coefficients below 0.10 indicate weak effects, values between 0.10 and 0.30 represent moderate effects, and values of 0.50 and above indicate strong effects of one variable on another [86]. As for the model fit indices listed in Table 7, it can be seen that all indices are within acceptable thresholds, indicating a good fit for the causal model.

**Table 7:** Goodness of fit measures of structural equation model of self-employment.

Index	Expected	Computed
Chi-square	–	144.500
df	–	35
Chi-square/df	5≥	4.12
GFI	0.9≤	0.969
NFI	0.9≤	0.945
CFI	0.9≤	0.956
RMSEA	0.06≥	0.028

#### 4.6.1. Causal effects of variables on the first-level mediating variable: Cinderella complex.

The causal effects of the independent variables on the Cinderella complex are summarized in Table 8 and illustrated in Fig 3. The results show that environmental factors exert a direct, positive, and significant influence on the Cinderella complex (β = 0.441, p = 0.0001), thereby supporting *H2.* This finding indicates that environmental factors play a crucial role in shaping and reinforcing the Cinderella complex. In particular, parenting styles within families, as well as the educational environments provided in schools and universities for girls, contribute to both the emergence and the intensification of this complex.

**Table 8:** Total and direct effects of research variables on Cinderella complex.

Variables	Standardized direct effects	Standardized total effects	Sig.
Structural factors	−0.064	−0.064	0.163
Environmental factors	0.441	0.441	0.0001
Cultural factors	−0.170	−0.170	0.0001

Furthermore, the analysis reveals that cultural factors have a direct, negative, and significant effect on the Cinderella complex (β = –0.170, p = 0.0001), providing support for *H1*. This outcome suggests that contemporary cultural conditions encourage women’s independence and reduce their inclination toward dependence on men. In contrast, structural factors show no significant direct effect on the Cinderella complex, leading to the rejection of *H3* (β = –0.064, p = 0.163).

#### 4.6.2. Causal effects of variables on the second-level mediating variables.

The results in Table 9 demonstrate that the Cinderella complex exerts a direct, negative, and significant effect on entrepreneurial enthusiasm (β = –0.214, p = 0.0001), confirming *H4.* This finding indicates that women with a stronger Cinderella complex—characterized by higher psychological dependency—exhibit lower levels of interest and enthusiasm toward entrepreneurship. Conversely, as the intensity of the Cinderella complex decreases and independence increases, women’s entrepreneurial enthusiasm rises. In addition, cultural factors indirectly influence entrepreneurial enthusiasm through their effect on the Cinderella complex (0.036). Environmental factors exert an indirect, negative effect (–0.094).

**Table 9:** Total, direct and indirect effects of research variables on entrepreneurial enthusiasm, self-efficacy and empowerment.

Mediator	Variables	Standardized direct effects	Standardized indirect effects	Standardized total effects	Sig.
Entrepreneurial enthusiasm	Cinderella complex	−0.214	–	−0.214	0.0001
Self-efficacy	Cinderella complex	−0.516	–	−0.516	0.0001
Empowerment	Cinderella complex	−0.171	−0.250	−0.421	0.0001
	Entrepreneurial enthusiasm	0.151	–	0.151	0.0001
	Self-efficacy	0.422	–	0.422	0.0001

Regarding self-efficacy, the Cinderella complex demonstrates a direct, negative, and significant effect (β = –0.516, p = 0.0001), thereby supporting *H6.* These results suggest that higher levels of the Cinderella complex diminish women’s self-efficacy. Cultural factors exert a positive indirect effect (0.088) via the Cinderella complex, while environmental factors influence self-efficacy indirectly and negatively (–0.228).

As shown in Fig 3, the results further indicate that the Cinderella complex has a direct, negative, and significant effect on empowerment (β = –0.171, p = 0.0001), confirming *H5.* This suggests that dependency is incompatible with empowerment; women with a higher Cinderella complex are less likely to feel empowered. Conversely, entrepreneurial enthusiasm has a direct, positive, and significant effect on empowerment (β = 0.151, p = 0.0001), confirming *H8.* This highlights that women who are more enthusiastic about entrepreneurship are more likely to achieve empowerment. Finally, self-efficacy has a direct, positive, and significant impact on empowerment (β = 0.422, p = 0.0001), confirming *H10,* showing that higher self-efficacy strongly enhances women’s empowerment. In terms of indirect effects, environmental factors negatively influence empowerment (–0.186) through their impact on the Cinderella complex. Cultural factors indirectly enhance empowerment (0.071) through their effect on the Cinderella complex.

#### 4.6.3. Causal effects of research variables on the dependent variable: Self-employment.

The results of Table 10 indicate that the Cinderella complex has a direct, negative, and significant effect on self-employment (β = –0.173, *p* = 0.0001), confirming *H7.* As a central factor in this study, the Cinderella complex reduces the likelihood of self-employment, suggesting that women who exhibit higher levels of dependency—stemming from fears of independence, avoidance of responsibility, and related psychological patterns—are less inclined to initiate entrepreneurial activities or establish their own businesses. Accordingly, creating conditions that prevent the emergence and intensification of the Cinderella complex among women is essential for fostering self-employment. Beyond its direct impact, the Cinderella complex also exerts an indirect negative effect on self-employment (–0.213). This occurs primarily through its adverse influence on entrepreneurial enthusiasm, empowerment, and self-efficacy.

**Table 10:** Total, direct and indirect effects of research variables on self-employment.

Variables	Standardized direct effects	Standardized indirect effects	Standardized total effects	Sig.
Cinderella complex	−0.173	−0.213	−0.386	0.0001
Entrepreneurial enthusiasm	0.575	0.004	0.579	0.0001
Self-efficacy	0.154	0.011	0.165	0.001
Empowerment	0.326	–	0.326	0.006

The findings further reveal that entrepreneurial enthusiasm has a direct, positive, and significant effect on self-employment (β = 0.575, *p* = 0.0001), thereby supporting *H9.* This demonstrates that women with higher entrepreneurial enthusiasm are more motivated to pursue self-employment, as enthusiasm propels them toward entrepreneurial action. Similarly, self-efficacy shows a direct, positive, and significant effect on self-employment (β = 0.154, *p* = 0.001), confirming *H11.* Women with stronger self-efficacy—expressed as greater confidence in their abilities—are more likely to establish self-employment ventures. In addition, self-efficacy indirectly influences self-employment through related pathways (0.011).

The analysis also highlights that empowerment exerts a direct, positive, and significant influence on self-employment (β = 0.326, *p* = 0.006), supporting *H12.* Women who are more socially engaged and exercise greater control and mastery over their lives demonstrate higher levels of empowerment, which increases their likelihood of pursuing self-employment. With respect to contextual variables, cultural factors indirectly and positively affect self-employment (0.066). In contrast, environmental factors negatively influence self-employment indirectly (–0.170) through their impact on the Cinderella complex and other mediating variables. Finally, structural factors exert an indirect effect on self-employment (0.025), primarily through their influence on entrepreneurial enthusiasm.

## 5. Discussion

The findings of this study highlight several important associations between psychological, socio-cultural, and structural factors and rural women’s engagement in self-employment. In line with theoretical expectations, the results indicate that the Cinderella complex is negatively associated with women’s self-employment. Women exhibiting higher levels of psychological dependency, avoidance of responsibility, and reliance on external validation tend to report lower levels of entrepreneurial engagement. This observation is consistent with psycho-social theories emphasizing the role of internalized social roles and self-perception in shaping behavior [87]. From the perspective of social cognitive theory, individuals’ beliefs about their capabilities and autonomy influence their goal-directed behavior [35]. Accordingly, women with stronger Cinderella complex traits may experience reduced confidence and a preference for avoiding responsibility, which are associated with lower self-reported entrepreneurial activity. Moreover, the Cinderella complex was linked to lower levels of entrepreneurial enthusiasm, suggesting that internalized dependency may constrain motivational states that support entrepreneurial aspirations.

Self-efficacy emerged as an important psychological correlate of self-employment. Women with higher self-efficacy tended to report greater engagement in entrepreneurial activities, highlighting the potential role of confidence and perceived competence in shaping women’s entrepreneurial intentions. This association aligns with Bandura’s theory, which emphasizes that self-efficacy influences persistence, goal-setting, and task-oriented behaviors [88]. In the current study, self-efficacy was also associated with empowerment, suggesting that women who perceive themselves as capable may feel more control over their personal and economic decisions, which in turn is linked to greater participation in self-employment. Similarly, entrepreneurial enthusiasm was positively associated with self-employment, indicating that women who report higher levels of motivation, interest, and affective engagement toward entrepreneurial activities are more likely to pursue self-employment. This relationship can be understood through the lens of SCT, where motivational states and outcome expectations influence goal-directed behaviors [89]. Enthusiasm also appeared to be related to empowerment, suggesting that motivational engagement may contribute to women’s perceived agency and proactive decision-making in entrepreneurial contexts.

Empowerment itself was positively associated with self-employment, reinforcing the notion that women who perceive greater control over resources and decision-making are more inclined to participate in entrepreneurial activities. This observation aligns with prior literature highlighting empowerment as a key enabler of women’s economic engagement and social agency [90,91].

In addition to psychological factors, the analysis highlighted the relevance of contextual variables. Cultural factors were indirectly linked to self-employment through their association with the Cinderella complex, suggesting that more supportive cultural norms are associated with lower dependency tendencies and, subsequently, higher entrepreneurial engagement. Structural factors, while not directly associated with self-employment, were indirectly linked through entrepreneurial enthusiasm, implying that access to education, resources, and social networks may foster motivational states that encourage women’s entrepreneurial activity. These findings are consistent with resource-based perspectives, which emphasize the role of access to tangible and intangible resources in supporting entrepreneurial engagement [92].

These findings suggest that psychological constructs such as the Cinderella complex, self-efficacy, entrepreneurial enthusiasm, and empowerment are meaningfully associated with women’s self-employment. However, given the cross-sectional nature of the data, it is important to interpret these associations cautiously. While the results are consistent with theoretical expectations, they do not allow for causal inferences. Future research employing longitudinal or experimental designs would be needed to establish directional or causal relationships among these variables.

This study underscores the importance of integrating psychological, socio-cultural, and structural perspectives when examining women’s entrepreneurial engagement in rural contexts. The findings highlight those interventions aimed at fostering self-efficacy, empowerment, and entrepreneurial motivation, alongside supportive cultural and structural conditions, may be associated with greater participation of women in self-employment.

## 6. Conclusion

This study has provided valuable insights into the psychological, socio-cultural, and environmental factors associated with self-employment among rural women. By examining the roles of the Cinderella complex, entrepreneurial enthusiasm, self-efficacy, and empowerment, it has become evident that these factors are meaningfully associated with the entrepreneurial behaviors and decisions of rural women. While cognitive and socio-cultural barriers such as the Cinderella complex present substantial challenges, there is also a clear opportunity to foster entrepreneurial enthusiasm and self-efficacy, which may serve as important enablers for self-employment.

The findings underscore the need for a comprehensive approach to address both the cognitive barriers and socio-cultural constraints that are linked to limited entrepreneurial potential. Women in rural areas, particularly in countries like Iran, often face systemic and psychological obstacles that hinder their entrepreneurial ambitions. However, these challenges are not insurmountable. Rather, they call for targeted interventions that can address these barriers while simultaneously supporting the development of empowerment, self-efficacy, and entrepreneurial motivation.

One of the key recommendations arising from this study is the urgent need for gender-sensitive policies that promote equality and provide women with equal opportunities in all aspects of life, particularly in business and entrepreneurship. Governments should prioritize initiatives that address legal and cultural barriers preventing women from accessing resources and pursuing entrepreneurial ventures. This includes removing discriminatory practices in the legal system, ensuring equal access to financial resources, and promoting fair representation of women in leadership roles. Furthermore, given the psychological constraints highlighted by the Cinderella complex, it is crucial to develop programs aimed at enhancing women’s mental and emotional resilience. Community-based initiatives that provide mentorship, leadership training, and psychological support can help women challenge traditional gender norms and gradually shift internalized dependency patterns, thereby supporting greater engagement in entrepreneurial activity.

Education and training are also critical to increasing entrepreneurial enthusiasm and self-efficacy. Investing in both formal and informal educational programs that focus on entrepreneurial skills, business management, and financial literacy can help rural women gain the tools they need to pursue and sustain their own businesses. Moreover, building strong support networks for rural women entrepreneurs is essential. These networks should facilitate the sharing of experiences, resources, and knowledge, enabling women to overcome the isolation that often characterizes rural settings. Collaborations with local businesses, NGOs, and community organizations can extend these networks and create opportunities for women to access broader markets, consistent with the observed associations between structural factors and entrepreneurial enthusiasm in our study. Lastly, media and public awareness campaigns can play a pivotal role in shifting societal perceptions and inspiring change. By showcasing successful female entrepreneurs and promoting the value of women’s economic independence, media outlets can help challenge stereotypes and encourage other women to pursue entrepreneurship. Highlighting the achievements of rural women can reinforce empowerment and self-efficacy, further supporting their motivation to engage in self-employment.

In conclusion, addressing the socio-psychological and structural factors associated with the self-employment potential of rural women is crucial not only for empowering women but also for fostering sustainable economic development in rural communities. By implementing these recommendations, countries like Iran can support the realization of untapped entrepreneurial potential among rural women, contributing to a more inclusive and resilient economy.

### 6.1. Research limitations

While this study provides valuable insights, it is important to acknowledge several limitations. First, the research is limited by its focus on a specific geographical area, particularly rural women in Iran. The contextual differences in gender roles, cultural norms, and economic conditions across different countries or regions could lead to variations in the factors that affect self-employment decisions among women. Furthermore, while the study includes several key psychological and socio-environmental factors, there may be other significant variables not considered here. For instance, external factors such as market conditions, access to technology, and the impact of social media on entrepreneurial activities were not directly addressed. Lastly, while the study emphasizes women’s psychological barriers and socio-cultural constraints, it does not explore the role of male counterparts, family dynamics, or male-dominated social structures in influencing women’s entrepreneurial aspirations.

### 6.2. Future directions

It would be beneficial to investigate how cultural and religious influences intersect with women’s entrepreneurial motivations. This could be done by comparing rural women in different regions across different cultural contexts globally. Such studies could examine the nuances of local norms, values, and religious beliefs in shaping women’s self-perception and entrepreneurial aspirations. Another promising direction for future research involves the role of men and family dynamics in supporting or hindering women’s entrepreneurial efforts. Understanding how men’s roles—whether as partners, fathers, or community leaders—interact with women’s entrepreneurial goals would add another layer of understanding to the socio-cultural context in which rural women operate. In patriarchal settings, the influence of male family members or male-dominated networks could either facilitate or constrain women’s participation in entrepreneurial activities. Additionally, exploring intervention strategies that can be practically applied to reduce psychological barriers, such as the Cinderella complex, and enhance self-efficacy and entrepreneurial enthusiasm among rural women, would be a critical area for applied research. These interventions could be developed into programs tailored to rural contexts, providing women with the resources, skills, and emotional support needed to pursue self-employment successfully.

Future studies could also examine the role of governmental policies and institutional initiatives aimed at fostering entrepreneurial efficacy among women. For example, national or local programs that provide training, access to credit, mentorship, and entrepreneurial networks could be evaluated for their effectiveness in enhancing women’s self-efficacy and reducing dependency-related psychological barriers. Furthermore, investigating urban versus rural differences would offer valuable insights into potential moderating effects of geographic and infrastructural factors on the pathways linking the Cinderella complex, self-efficacy, and entrepreneurial behavior. Such comparative studies could reveal whether intervention strategies need to be tailored differently across contexts and could inform policy design to more effectively promote women’s self-employment.

## Supporting information

S1 Appendix CFA details for *Cinderella complex.* (DOCX)

S2 Appendix CFA details for *empowerment.* (DOCX)

S3 Appendix CFA details for *environmental factors.* (DOCX)

S4 Appendix De-identified dataset supporting the findings of this study. (XLSX)
