# Supplementary material for: Breaking dependency: The Cinderella complex and barriers to self-employment among rural women in Iran
Source: PLoS One. 2026 Feb 25;21(2):e0337652. doi: 10.1371/journal.pone.0337652 (PMC12935258; doi:10.1371/journal.pone.0337652)
Supplement: S1 Appendix — (DOCX) [file pone.0337652.s001.docx]

**S1 Appendix-** CFA details for *Cinderella complex*


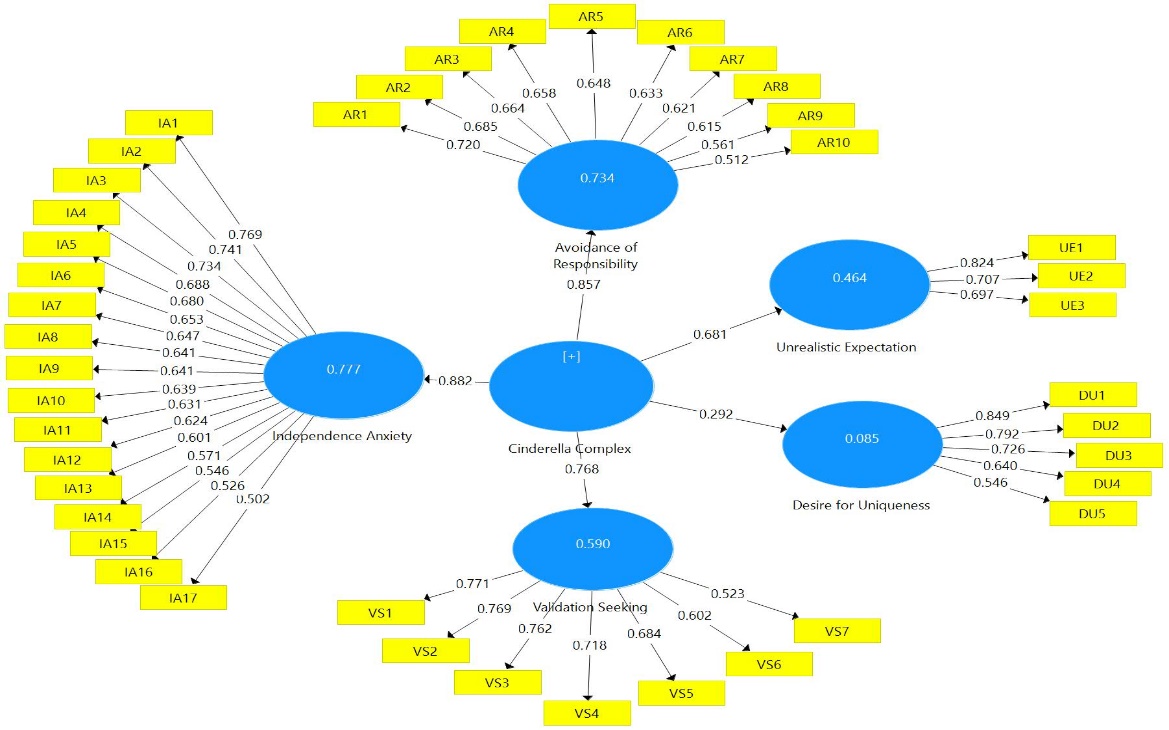


| AVE | CR | Cronbach Alpha | Factor Loading | Items | Code | Components |
| --- | --- | --- | --- | --- | --- | --- |
| 0.511 | 0.921 | 0.909 | 0.769 | Viewing marriage as the only path for support and protection of women | IA1 | Independence Anxiety  (IA) |
|  |  |  | 0.741 | Preference for being with a spouse rather than being alone | IA2 |  |
|  |  |  | 0.734 | Quitting a job and relying on the spouse's income | IA3 |  |
|  |  |  | 0.688 | Women’s need for a prince on a white horse to achieve a better life | IA4 |  |
|  |  |  | 0.680 | A woman's sacrifice for her husband's success and not being left alone in old age | IA5 |  |
|  |  |  | 0.653 | Giving up the dream of having an independent job due to marriage | IA6 |  |
|  |  |  | 0.647 | Acceptance of the husband or father as the main decision-makers and the lack of involvement of women in family decision-making | IA7 |  |
|  |  |  | 0.641 | Reducing women's career aspirations to preserve feminine qualities | IA8 |  |
|  |  |  | 0.641 | Men make better decisions than women due to being better educated | IA9 |  |
|  |  |  | 0.639 | Acceptance of the man's role as the protector and the woman as the one in need of protection in human relationships | IA10 |  |
|  |  |  | 0.631 | The influential role of men in fulfilling women's desires for a better and more comfortable life | IA11 |  |
|  |  |  | 0.624 | The ease and stress-free nature of household and family tasks compared to outside work | IA12 |  |
|  |  |  | 0.601 | Giving up a beloved job to save the relationship with a spouse | IA13 |  |
|  |  |  | 0.571 | The importance of maintaining feminine attractiveness and a woman's obedience over her independence | IA14 |  |
|  |  |  | 0.546 | Giving up on dreams for the sake of a loved one, even if it is the wrong decision | IA15 |  |
|  |  |  | 0.526 | Delegating future planning to others and freeing oneself from confusion in decision-making | IA16 |  |
|  |  |  | 0.502 | The type of upbringing of women from childhood to obey the male family members (father, brother, husband) | IA17 |  |
| 0.502 | 0.870 | 0.833 | 0.720 | A strong dependence on others in carrying out activities and responsibilities, along with a deep fear of the idea of being alone and working independently | AR1 | Avoidance of Responsibility  (AR) |
|  |  |  | 0.685 | Worry and stress in managing new matters alone | AR2 |  |
|  |  |  | 0.664 | Not completing all activities despite having the ability to do so | AR3 |  |
|  |  |  | 0.658 | Emotional distress when being abandoned by the counterpart and an inability to fulfill life responsibilities | AR4 |  |
|  |  |  | 0.648 | Severe anxiety and stress in fulfilling any responsibility successfully, instead of feeling happy about accomplishing them | AR5 |  |
|  |  |  | 0.633 | Striving to keep others satisfied in carrying out activities in order to retain them under any circumstances | AR6 |  |
|  |  |  | 0.621 | Being dependent in performing tasks and responsibilities | AR7 |  |
|  |  |  | 0.615 | Leaving tasks unfinished due to the feeling of incapacity to complete them | AR8 |  |
|  |  |  | 0.561 | The need for others' support to undertake a major activity or new project | AR9 |  |
|  |  |  | 0.512 | The inability to express emotions openly, leading to missed opportunities as a result | AR10 |  |
| 0.555 | 0.788 | 0.700 | 0.824 | Expecting a partner to constantly indulge and pamper, similar to how parents treated them during childhood | UE1 | Unrealistic Expectation (UE) |
|  |  |  | 0.707 | The obligation for a woman to create a calm work environment for her husband | UE2 |  |
|  |  |  | 0.697 | Choosing a man who takes on all the responsibilities of life | UE3 |  |
| 0.517 | 0.839 | 0.776 | 0.849 | The feeling of having the ability to cope with the complexities and challenges of life and being strong in problem-solving | DU1 | Desire for Uniqueness (DU) |
|  |  |  | 0.792 | Pride in oneself and not caring about the judgment of others | DU2 |  |
|  |  |  | 0.726 | Perceiving oneself as a person with high self-confidence | DU3 |  |
|  |  |  | 0.640 | The ability to guide friends and family through difficult life situations | DU4 |  |
|  |  |  | 0.546 | The willingness to take on the role of a group leader | DU5 |  |
| 0.584 | 0.866 | 0.817 | 0.771 | Exaggerating personal needs to gain the support of others | VS1 | Validation Seeking (VS) |
|  |  |  | 0.769 | Fear of losing the love and support of those around | VS2 |  |
|  |  |  | 0.762 | Desire to be the center of attention | VS3 |  |
|  |  |  | 0.718 | Avoiding activities due to the fear of not receiving attention or appreciation from others | VS4 |  |
|  |  |  | 0.684 | Dissatisfaction with the feedback from others regarding assigned tasks | VS5 |  |
|  |  |  | 0.602 | Easily and quickly persuaded by others | VS6 |  |
|  |  |  | 0.523 | Feeling weak in abilities and skills, and lacking initiative in activities | VS7 |  |

| Components | Avoidance of Responsibility | Desire for Uniqueness | Independence Anxiety | Unrealistic Expectation | Validation Seeking |
| --- | --- | --- | --- | --- | --- |
| Avoidance of Responsibility | 0.708 |  |  |  |  |
| Desire for Uniqueness | 0.339 | 0.719 |  |  |  |
| Independence Anxiety | 0.597 | 0.114 | 0.714 |  |  |
| Unrealistic Expectation | 0.512 | 0.041 | 0.566 | 0.745 |  |
| Validation Seeking | 0.600 | 0.257 | 0.478 | 0.512 | 0.764 |
