# Supplementary material for: Breaking dependency: The Cinderella complex and barriers to self-employment among rural women in Iran
Source: PLoS One. 2026 Feb 25;21(2):e0337652. doi: 10.1371/journal.pone.0337652 (PMC12935258; doi:10.1371/journal.pone.0337652)
Supplement: S2 Appendix — (DOCX) [file pone.0337652.s002.docx]

**S2 Appendix-** CFA details for *Empowerment*


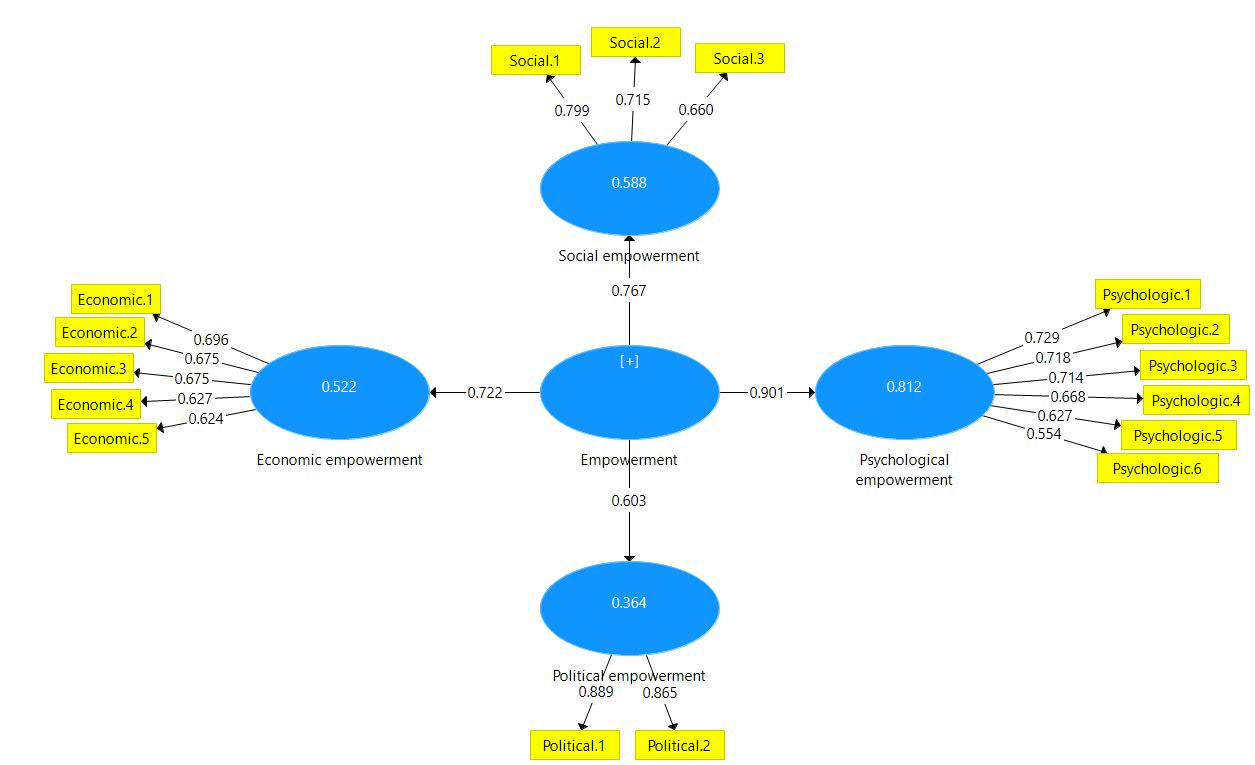


| AVE | CR | Cronbach Alpha | Factor Loading | Items | Code | Components |
| --- | --- | --- | --- | --- | --- | --- |
| 0.536 | 0.794 | 0.700 | 0.696 | The ability to earn money and increase household income | Economic1 | Economic |
|  |  |  | 0.675 | The ability to repay monthly loan installments for oneself and one’s family | Economic2 |  |
|  |  |  | 0.675 | Having sufficient income to cover basic living expenses | Economic3 |  |
|  |  |  | 0.627 | The ability to save a portion of monthly income | Economic4 |  |
|  |  |  | 0.624 | The ability to manage personal assets such as a house and a car | Economic5 |  |
| 0.528 | 0.769 | 0.748 | 0.799 | Awareness of societal issues | Social1 | Social |
|  |  |  | 0.715 | Recognizing the importance of collaboration and teamwork | Social2 |  |
|  |  |  | 0.660 | Unrestricted ability to leave the home for employment purposes | Social3 |  |
| 0.550 | 0.830 | 0.753 | 0.729 | Enjoying freedom of action in personal and decision-making choices | Psychological1 | Psychological |
|  |  |  | 0.718 | Exhibiting strong creativity and innovation in decision-making processes | Psychological2 |  |
|  |  |  | 0.714 | The capacity to influence others and initiate change within one's environment | Psychological3 |  |
|  |  |  | 0.668 | Effectively communicating with others | Psychological4 |  |
|  |  |  | 0.627 | Having the necessary academic and practical skills to execute professional tasks | Psychological5 |  |
|  |  |  | 0.554 | Having comparable capabilities relative to one's peers | Psychological6 |  |
| 0.769 | 0.869 | 0.700 | 0.889 | Awareness of the country's political issues | Political1 | Political |
|  |  |  | 0.865 | Engagement in political discussions within the family | Political2 |  |

| Components | Economic Empowerment | Social Empowerment | Psychological Empowerment | Political Empowerment |
| --- | --- | --- | --- | --- |
| Economic Empowerment | 0.732 |  |  |  |
| Social Empowerment | 0.424 | 0.726 |  |  |
| Psychological Empowerment | 0.490 | 0.601 | 0.741 |  |
| Political Empowerment | 0.240 | 0.352 | 0.465 | 0.877 |
