# Supplementary material for: Breaking dependency: The Cinderella complex and barriers to self-employment among rural women in Iran
Source: PLoS One. 2026 Feb 25;21(2):e0337652. doi: 10.1371/journal.pone.0337652 (PMC12935258; doi:10.1371/journal.pone.0337652)
Supplement: S3 Appendix — (DOCX) [file pone.0337652.s003.docx]

**S3 Appendix-** CFA details for *Environmental Factors*


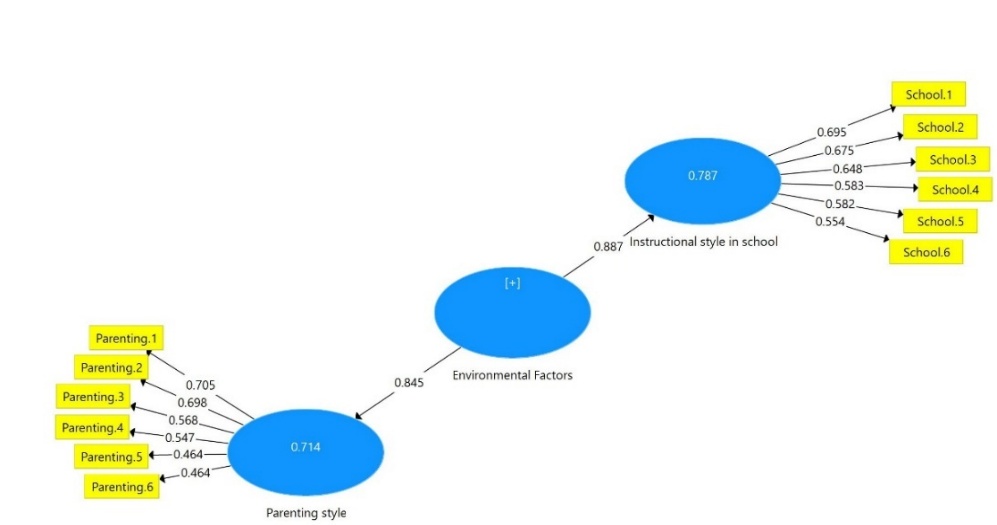


| AVE | CR | Cronbach Alpha | Factor Loading | Items | Code | Components |
| --- | --- | --- | --- | --- | --- | --- |
| 0.500 | 0.750 | 0.700 | 0.705 | The risk of girls becoming uncontrollable and defiant as a result of continuing their education | Parenting1 | Parenting style |
|  |  |  | 0.698 | A higher emphasis on investing in the education of boys | Parenting2 |  |
|  |  |  | 0.568 | Greater emphasis on marriage over education and employment for girls | Parenting3 |  |
|  |  |  | 0.547 | Gender inequality and increased freedom for boys within rural families | Parenting4 |  |
|  |  |  | 0.464 | Rural families' stronger preference for having sons | Parenting5 |  |
|  |  |  | 0.464 | Limited family support for the education of girls in rural areas | Parenting6 |  |
| 0.591 | 0.792 | 0.787 | 0.695 | The lack of equal educational opportunities for girls compared to boys | School1 | Instructional style in school |
|  |  |  | 0.675 | Boys growing up to be more independent during their schooling compared to girls | School2 |  |
|  |  |  | 0.648 | The increased restrictions and challenges encountered by girls in educational settings, compared to boys | School3 |  |
|  |  |  | 0.583 | The lack of academic success among girls in comparison to boys | School4 |  |
|  |  |  | 0.582 | A higher number of field trips and recreational outings for boys in comparison to girls | School5 |  |
|  |  |  | 0.554 | Greater freedom afforded to boys during their schooling | School6 |  |

| Components | Parenting style | Instructional style in school |
| --- | --- | --- |
| Parenting style | 0.707 |  |
| Instructional style in school | 0.503 | 0.768 |
